# Integrated In‐Memory Sensor and Computing of Artificial Vision Based on Full‐vdW Optoelectronic Ferroelectric Field‐Effect Transistor

**DOI:** 10.1002/advs.202305679

**Published:** 2023-11-29

**Authors:** Peng Wang, Jie Li, Wuhong Xue, Wenjuan Ci, Fengxian Jiang, Lei Shi, Feichi Zhou, Peng Zhou, Xiaohong Xu

**Affiliations:** ^1^ Key Laboratory of Magnetic Molecules and Magnetic Information Materials of Ministry of Education & School of Chemistry and Materials Science Shanxi Normal University Taiyuan 030031 China; ^2^ School of Microelectronics Southern University of Science and Technology Shenzhen 518000 China; ^3^ ASIC & System State Key Lab School of Microelectronics Fudan University Shanghai 200433 China

**Keywords:** full‐vdW ferroelectric field effect transistor, multilevel memory, neuromorphic vision system, sensing‐memory‐computing

## Abstract

The development and application of artificial intelligence have led to the exploitation of low‐power and compact intelligent information‐processing systems integrated with sensing, memory, and neuromorphic computing functions. The 2D van der Waals (vdW) materials with abundant reservoirs for arbitrary stacking based on functions and enabling continued device downscaling offer an attractive alternative for continuously promoting artificial intelligence. In this study, full 2D SnS_2_/h‐BN/CuInP_2_S_6_ (CIPS)‐based ferroelectric field‐effect transistors (Fe‐FETs) and utilized light‐induced ferroelectric polarization reversal to achieve excellent memory properties and multi‐functional sensing‐memory‐computing vision simulations are designed. The device exhibits a high on/off current ratio of over 10^5^, long retention time (>10^4^ s), stable cyclic endurance (>350 cycles), and 128 multilevel current states (7‐bit). In addition, fundamental synaptic plasticity characteristics are emulated including paired‐pulse facilitation (PPF), short‐term plasticity (STP), long‐term plasticity (LTP), long‐term potentiation, and long‐term depression. A ferroelectric optoelectronic reservoir computing system for the Modified National Institute of Standards and Technology (MNIST) handwritten digital recognition achieved a high accuracy of 93.62%. Furthermore, retina‐like light adaptation and Pavlovian conditioning are successfully mimicked. These results provide a strategy for developing a multilevel memory and novel neuromorphic vision systems with integrated sensing‐memory‐processing.

## Graphical abstract

A novel multi‐functional neuromorphic visual system with optoelectronic synergy based on SnS_2_/BN/CuInP_2_S_6_ full van der Waals ferroelectric field‐effect transistor is reported. The device demonstrates a high switching ratio of 10^5^, multilevel storage states of 128 (7 bits), excellent synaptic plasticity, and an image recognition accuracy of 93.62% based on reservoir computing.

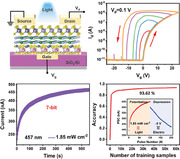

## 1 Introduction

As artificial intelligence (AI) technology is gradually infiltrating into various applications, the market demand for computing power is rapidly growing.^[^ ^1^, ^2^, ^3^ ^]^ Owing to the physical separation of memory and computing in von Neumann architecture,^[^ ^4^, ^5^ ^]^ the energy efficiency problem caused by frequent data interactions has become increasingly critical, and the chip industry must urgently innovate at the level of the underlying computing architecture.^[^ ^6^, ^7^, ^8^, ^9^ ^]^ In‐memory computing is regarded as the core of AI innovation, in which the “memory wall” and “power wall” caused by data handling are avoided, and data parallelism and energy efficiency are significantly improved.^[^ ^10^, ^11^, ^12^ ^]^ Considering the rapid development of novel artificial vision systems, new requirements have been proposed for novel vision devices with real‐time acquisition, efficient processing, and timely decision‐making regarding external environment information.^[^ ^11^, ^13^, ^14^, ^15^, ^16^, ^17^ ^]^ The conventional artificial vision system physically separates the sensors into memory and computing units, thereby generating unstructured and redundant data during the processing of the image sensor node. Inspired by the human retina,^[^ ^18^ ^]^ a neuromorphic vision sensor senses optical signals, stores signals, and preprocesses information can mimic the function of the human retina.^[^ ^19^, ^20^, ^21^, ^22^ ^]^ The neuromorphic visual sensor architecture with integrated sensing‐memory‐computing facilitates avoiding the power consumption bottleneck of data handling in the von Neumann architecture.^[^ ^14^, ^15^, ^16^ ^]^ This would improve the overall efficiency and reduce the computational delay as well as redundant data storage, from sensing to computing in the image sensor.

A neuromorphic visual sensor with integrated sensing‐memory‐computing was attempted for implementation in the popular Fe‐FET. This is because ferroelectric ordering can be controlled by electricity and light,^[^ ^23^, ^24^, ^25^, ^26^ ^]^ modulating the carrier concentration of the semiconductors, and thereby adjusting their electron transport and optoelectronic properties.^[^ ^24^, ^27^ ^]^ For example, Tong et al.^[^ ^15^ ^]^ proposed a new architecture for integration of sensing‐memory‐computing by coupling semiconductor materials with conventional ferroelectric materials to emulate human retinal functions. Cui et al.^[^ ^28^ ^]^ reported a conventional ferroelectric photovoltage sensor network with integrated sensing‐memory‐computing, achieving zero‐energy image sensing and real‐time processing, and providing a feasible technical route for developing highly‐robust, high‐speed, and low‐energy machine vision systems. However, conventional ferroelectric materials have limitations such as reduced long‐range Coulomb coupling and strengthened depolarized electrostatic fields as the thickness decreases to several or tens of nanometers,^[^ ^29^, ^30^ ^]^ which significantly limits device miniaturization and high‐density integration.^[^ ^31^ ^]^ Furthermore, interface issues, such as surface reconstructions and defect states,^[^ ^32^, ^33^ ^]^ occur during the epitaxial growth of most conventional ferroelectrics on silicon owing to lattice mismatch, leading to their performance degradation and a poor reliability.^[^ ^34^ ^]^ These issues can be averted with a clean van der Waals (vdW) surface without the surface dangling bonds of 2D ferroelectrics,^[^ ^35^, ^36^ ^]^ which enables lattice mismatch for integration with most materials, including silicon. This demonstrates that 2D vdW ferroelectrics have a rich reservoir of arbitrarily stacked artificial heterostructures based on functions that enable continued device miniaturization.^[^ ^37^, ^38^, ^39^, ^40^ ^]^ Therefore, the full 2D Fe‐FET may be a potential solution to eliminate interface problems and achieve higher performance of sensing‐memory‐computing for compact multi‐functional neuromorphic vision system.

In this study, we designed and constructed a full vdWH Fe‐FET with uniform and smooth high‐quality interfaces using SnS_2_, h‐BN, and CuInP_2_S_6_ (CIPS). At the microscopic level, ferroelectric polarization switching induced by light and electric fields was observed, which modulates the electron transport and optoelectronic properties of the semiconductor. The device exhibited a high on/off ratio of over 10^5^, long retention time (>10^4^ s), stable cyclic endurance (>350 cycles), and 128 multilevel current states (7‐bit). The sensing, memory, and neuromorphic computing functions were implemented on a single device. Fundamental synaptic behaviors such as paired‐pulse facilitation (PPF), short‐term plasticity (STP), long‐term plasticity (LTP), long‐term potentiation, and long‐term depression were successfully simulated. Retina‐like adaptation to light and Pavlovian conditioning were also demonstrated. The implementation of the aforementioned functions stems from both light‐ and electricity‐controlled ferroelectric switching to modulate the electron transport and optoelectronic properties of the SnS_2_ semiconductor. A fully ferroelectric optoelectronic reservoir computing (RC) system was constructed using optical STP features for the reservoir layer and electrical long‐term potentiation and depression features for the fully connected layer. The RC‐based image recognition achieved a high recognition accuracy of 93.62%. This study demonstrates that the full 2D Fe‐FET can provide a new opportunity for the development of excellent multilevel memory and compact neuromorphic visual systems with integrated high‐performance sensing‐memory‐computing functions.

## 2 Results and Discussion

**Figure 1:**
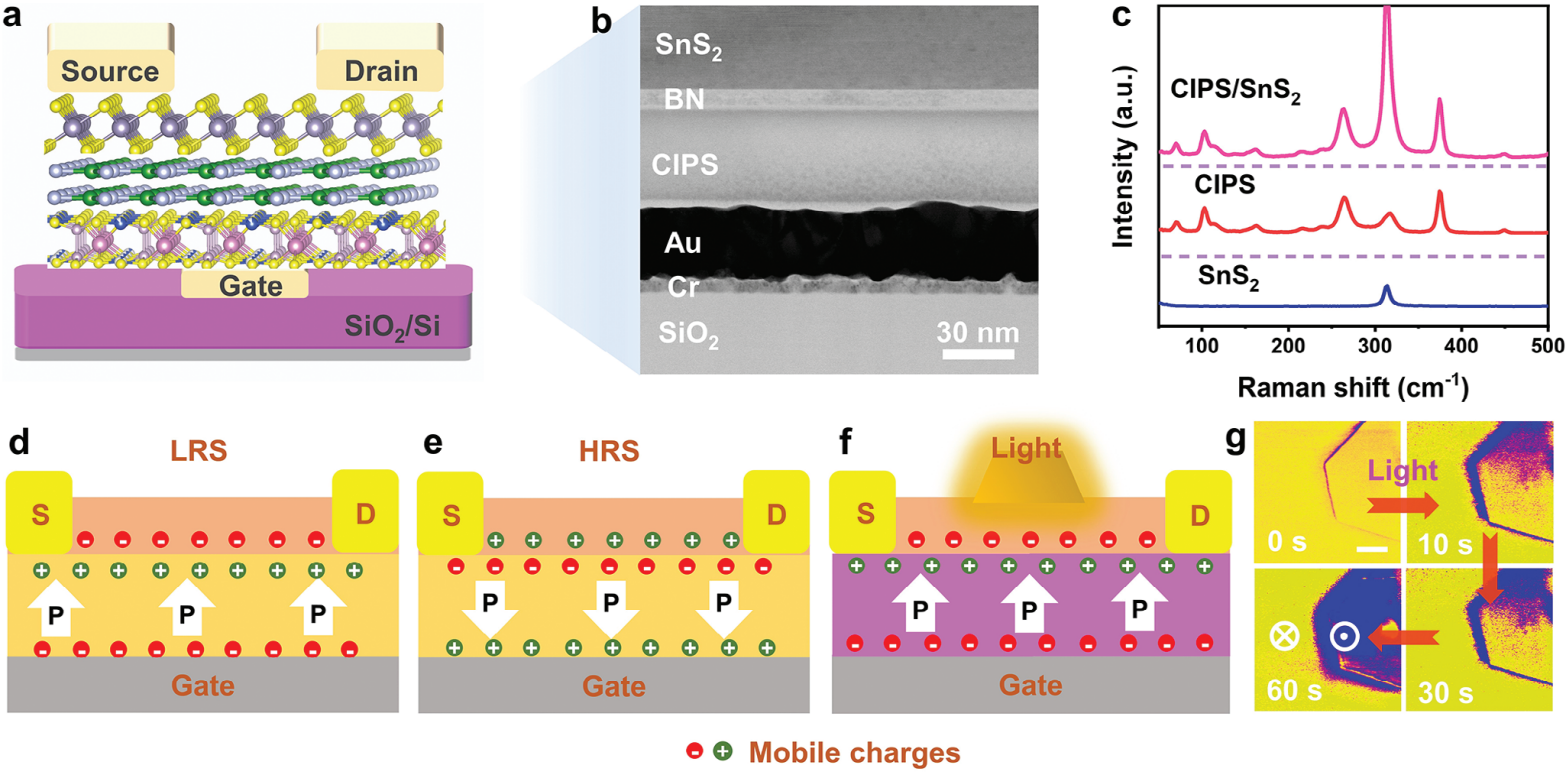
Structure and working mechanism of the SnS_2_/h‐BN/CIPS‐based Fe‐FET. a) Schematic of the three‐terminal vdWH Fe‐FET device. b) Cross‐sectional TEM image of the device. c) Raman spectra of SnS_2_, CIPS, and SnS_2_/CIPS heterojunction at room temperature. Mechanism of the d) upward and e) downward polarization states regulating the carrier concentration in the channel by applying positive and negative gate voltages. When polarization is upward, the SnS_2_ channel layer is in the electron accumulation state; otherwise, the channel layer is in the electron depletion state. f) Schematic of the light‐induced ferroelectric polarization reversal of CIPS nanosheets underneath the multilayer SnS_2_. g) PFM phase diagrams of the light‐induced CIPS ferroelectric domain evolution as a function of the exposure time (457 nm, 5 mW cm^−2^). Scale bar: 2 µm.

**Figure** **1**a presents a schematic of our designed van der Waals heterostructure (vdWH) Fe‐FET memory device, consisting of vertically stacked SnS_2_/h‐BN/CIPS vdWH on a SiO_2_/Si substrate of pre‐deposited Au electrodes, where SnS_2_, h‐BN, CIPS and Au serve as the channel layer, tunnel layer, control‐gate ferroelectric dielectric, and control gate, respectively. Pre‐deposited Au electrodes can prevent the pollution of organic solvents and the implantation of metal ions during evaporation, thus ensuring a clean and tidy interface. Figure S1 (Supporting Information) presents an optical image of a typical device in which one CIPS (marked by the black dashed line) is directly placed on the control gate electrode, followed by the sequential stacking of h‐BN (marked by the blue dashed line) and SnS_2_ (marked by the red dashed line). Further details regarding the materials and device fabrication processes can be found in the Experimental Section. The thicknesses of the SnS_2_, BN, and CIPS nanosheets measured by atomic force microscopy (AFM) were ≈23.6, 11.5, and 61.5 nm respectively (Figure S2, Supporting Information). The cross‐sectional high‐resolution transmission electron microscopy (TEM) images of the vdWH Fe‐FET devices are shown in Figure 1b. The layered interfaces between the different functional components of the as‐fabricated devices were clean and uniform, demonstrating the high‐quality interfaces of the devices. These results are critical for the achieving the following excellent performances.^[^ ^41^ ^]^ Figure 1c presents the Raman spectra of the SnS_2_, CIPS, and SnS_2_/CIPS heterostructures at room temperature. The spectrum of SnS_2_ (blue line) demonstrates the most intense dominant peak at 314 cm^−1^, corresponding to the A_1g_ mode.^[^ ^42^, ^43^ ^]^ CIPS Raman peaks appear at 75 and 315 cm^−1^ owing to the presence of cations (Cu^I^ and In^III^), the peaks at 100 cm^−1^ correspond to the anion (P_2_S_6_^4−^) vibrations, and the other observed Raman peaks sufficiently match with previous studies regarding CIPS.^[^ ^38^, ^39^, ^44^, ^45^ ^]^ All the peaks of CIPS and SnS_2_ were present in the vdWH region (pink line). No apparent peak shift was observed, indicating the high quality of the heterojunction region after the dry transfer process. To utilize photo‐induced ferroelectric polarization switching for achieving excellent multilevel memory and multi‐functional sensing‐memory‐computing vision simulations, the UV‐visible‐near‐infrared (UV‐vis‐NIR) absorption spectra of the SnS_2_ nanosheets and the optoelectronic ferroelectric polarization switching of CIPS were investigated. An absorption edge appeared at ≈521 nm; therefore, the band gap of SnS_2_ was estimated to be ≈2.38 eV by using the relationship between the absorption coefficient and incident photon energy (Figure S3b, Supporting Information). The ferroelectricity of CIPS was investigated using noninvasive piezoresponse force microscopy (PFM).^[^ ^44^ ^]^ As shown in Figure S4a (Supporting Information), the PFM phase image of the CIPS nanosheet after writing box‐in‐box patterns by applying a ±7 V bias reveals a strong phase contrast of ≈180°, corresponding to *P*_up_ and *P*_down_, respectively. The PFM amplitude signal obtained in Figure S4b (Supporting Information) indicates robust and intrinsic ferroelectric behavior. The corresponding well‐defined butterfly loops and 180° phase switch further confirm the robust ferroelectric polarization in the CIPS nanosheet (Figure S4c, Supporting Information). Furthermore, light‐induced ferroelectric switching in an Fe‐FET device was studied using the light‐assisted PFM measurement. The polarization state of the CIPS was first set to the downward state by a 7 V bias. After applying light pulses (457 nm, 5 mW cm^−2^) with different widths (10, 30, and 60 s), the polarization of the CIPS covered by SnS_2_ was gradually switched to the upward state as the illumination time increased, whereas the polarization direction of the bare CIPS remained unchanged (Figure 1g). The light‐controlled ferroelectric polarization switching can be explained by the interaction between the photogenerated charges in SnS_2_ and ferroelectric polarization charges in CIPS, according to the recently proposed theory.^[^ ^24^, ^26^, ^27^ ^]^ Due to the fact that the SnS_2_/CIPS heterojunction is essentially asymmetric with a preferential upward polarization, and therefore an upward built‐in electric field (*E*_bi_) exists. When the device is exposed to light, the photogenerated charge compensates the initially distributed interfacial charge, which results in *E*_bi_ switching the downward polarization to upward. A schematic of the mechanism is shown in Figure 1d–f. When the ferroelectric polarization of the CIPS is downward (*P*_down_), the electrons of the SnS_2_ channel are depleted, resulting in a high‐resistance state (HRS) (Figure 1e). Conversely, the upward polarization state causes the accumulation of electrons in the SnS_2_ channel and induces a low‐resistance state (LRS) (Figure 1d,f).

**Figure 2:**
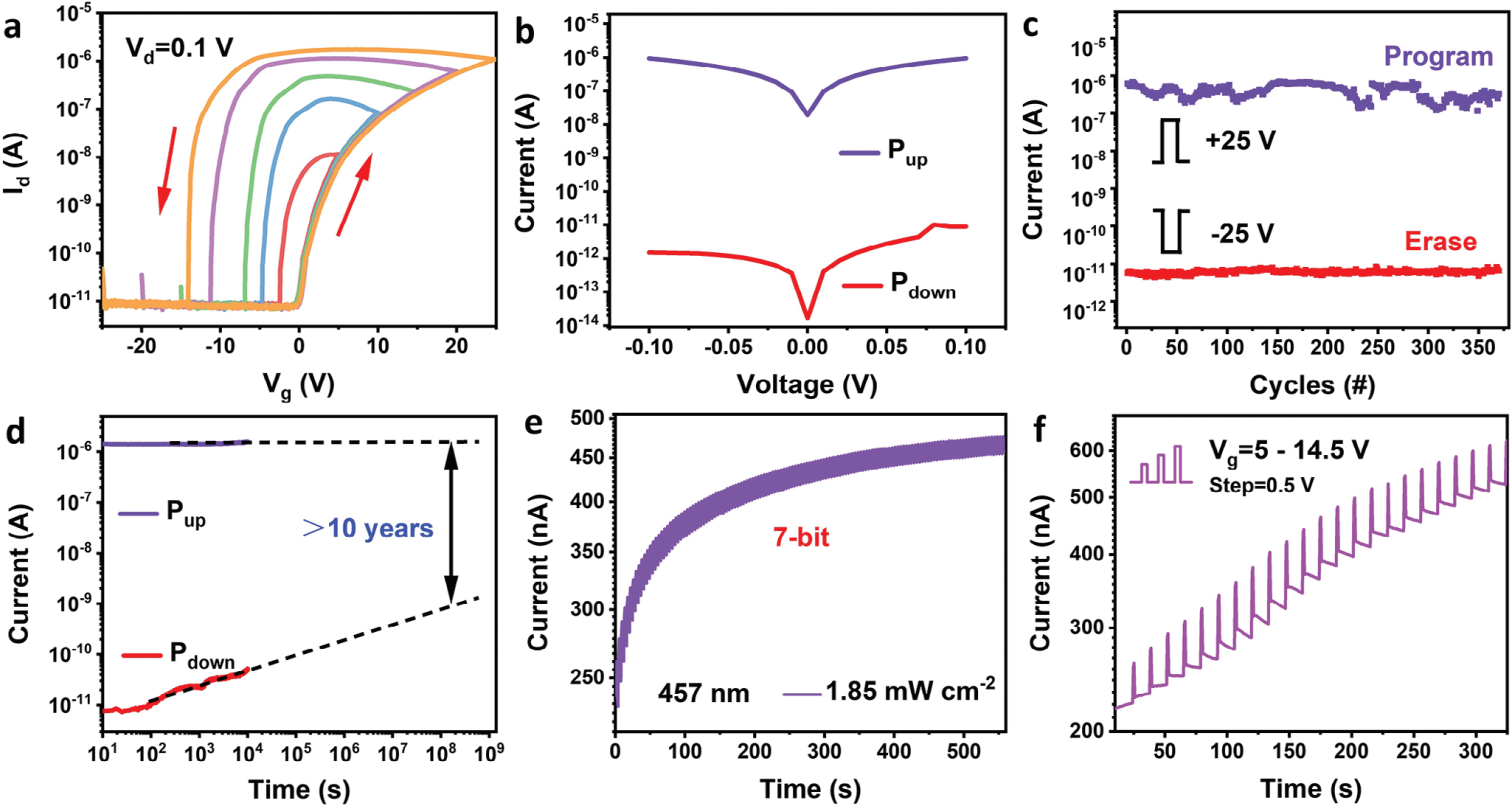
Memory proprieties of the SnS_2_/h‐BN/CIPS‐based Fe‐FET. a) Transfer characteristics under different gate voltage scanning ranges (from ±5 to ±25 V, step: 5 V; *V*_ds_ = 0.1 V). b) The current characteristics of the device after applying a ±25 V poling electric pulse (width: 10 s). The device exhibits a large memory window and on/off ratio up to 10^5^. c) Endurance characteristics of the device for over 350 P/E cycles. d) The retention performances of HRS (*P*_down_) and LRS (*P*_up_) for the device. Multilevel memory characteristics under e) periodic light pulses (457 nm, 1.85 mW cm^−2^, width: 1.5 s) and f) different electrical pulse amplitudes (from 5 to 14.5 V, step: 0.5 V, width: 0.1 s). The device current was read at 0.1 V for all experiments.

First, the memory performance reliability of the SnS_2_/BN/CIPS‐based vdWHs Fe‐FET was evaluated. The device displayed a low leakage current (Figure S5a, Supporting Information), which is crucial for achieving an excellent memory performance. **Figure** **2**a presents the transfer curves obtained using different gate voltages (*V*_g_) at a drain bias (*V*_d_) of 0.1 V, where the transfer curves exhibit *n*‐type semiconductor characteristics. When *V*_g_ is scanned from −25 to 25 V, a high on/off current ratio of 10^5^ and large memory window of 18.4 V are obtained. As the *V*_g_ scan range increases, the memory window linearly increases (Figure S5b, Supporting Information). Note, the forward and reverse transfer curve sweeps exhibit an anticlockwise hysteresis that is different from the previously reported clockwise hysteresis in *n*‐type channels.^[^ ^30^, ^38^, ^39^, ^41^, ^46^ ^]^ The anticlockwise hysteresis can be attributed to the ferroelectric polarization switching of the CIPS.^[^ ^47^ ^]^ Figure 2b shows the output characteristics after applying a *V*_g_ pulse to switch the direction of ferroelectric polarization. When applying a negative gate voltage (−25 V, 10 s), the polarization of CIPS was turned downward and the channel current decreased to 10^−11^ A. Conversely, when a positive gate voltage (+25 V, 10 s) was applied, the polarization of CIPS was upward and the channel current increased to 10^−6^ A. In addition, endurance and retention characteristics were determined to be important indicators of the memory performance. Figure 2c shows the cyclic program/erase (P/E) endurance at a *V*_g_ pulse (±25 V, 10 s) and reading voltage of 0.1 V, clearly demonstrating that both program and erase states remain nearly unchanged even after over 350 cycles. The reliable retention of is demonstrated in Figure 2d; the currents of the HRS and LRS exhibited negligible degradation even after 10^4^ s, and the current ratio is expected to be maintained at 10^3^ after 10 years. These excellent retention and durability properties may be attributed to the ability of the defect‐free h‐BN to improve the interfacial quality by suppressing leakage current and ion diffusion.^[^ ^39^, ^47^ ^]^ These results confirm that the device exhibits an outstanding memory performances.

This large P/E current ratio is favorable for achieving multilevel memory. Subsequently, we investigated the dynamic memory modulated by light and voltage pulse excitations. The periodic light pulses (1.85 mW cm^−2^, 1.5 s) were employed to program the device current after applying a gate voltage of −10 V. Notably, the channel current significantly increased with different current levels by applying 128 light pulses (Figure 2e). The corresponding partially enlarged view of Figure 2e is shown in Figure S6 (Supporting Information). The 128 effective storage states (7‐bit) exhibited in the device are higher than those previously reported for photoelectric memories.^[^ ^48^, ^49^, ^50^, ^51^, ^52^, ^53^, ^54^, ^55^ ^]^ This light‐excited multilevel memory is attributed to the dose of light irradiation that controls the concentration of photogenerated carriers in SnS_2_ to regulate the ferroelectric polarization state, causing a gradual accumulation of current (the detailed mechanism is shown in Figure S7, Supporting Information). Furthermore, the results of the exposure time‐ and intensity‐dependent output current with a light pulse (457 nm) demonstrate that the channel currents also increase significantly as the exposure time or light intensity increase (Figure S8, Supporting Information). The current did not return to its initial state when the light pulse was removed, further demonstrating the potential of the device use in multilevel memory. In addition to light stimulation, dynamic multilevel memory behavior can be modulated via the electric pulse amplitude (Figure 2f) and time (Figure S9, Supporting Information). When the applying electric pulse amplitude (time) was increased from 5 to 14.5 V (from 0.1 to 1.3 s) with a step of 0.5 V (0.1 s), the device current gradually increased, demonstrating the multilevel memory states. These results clearly indicate that the constructed SnS_2_/h‐BN/CIPS‐based Fe‐FET is suitable for applications in high‐density storage and multi‐functional neuromorphological vision systems.

**Figure 3:**
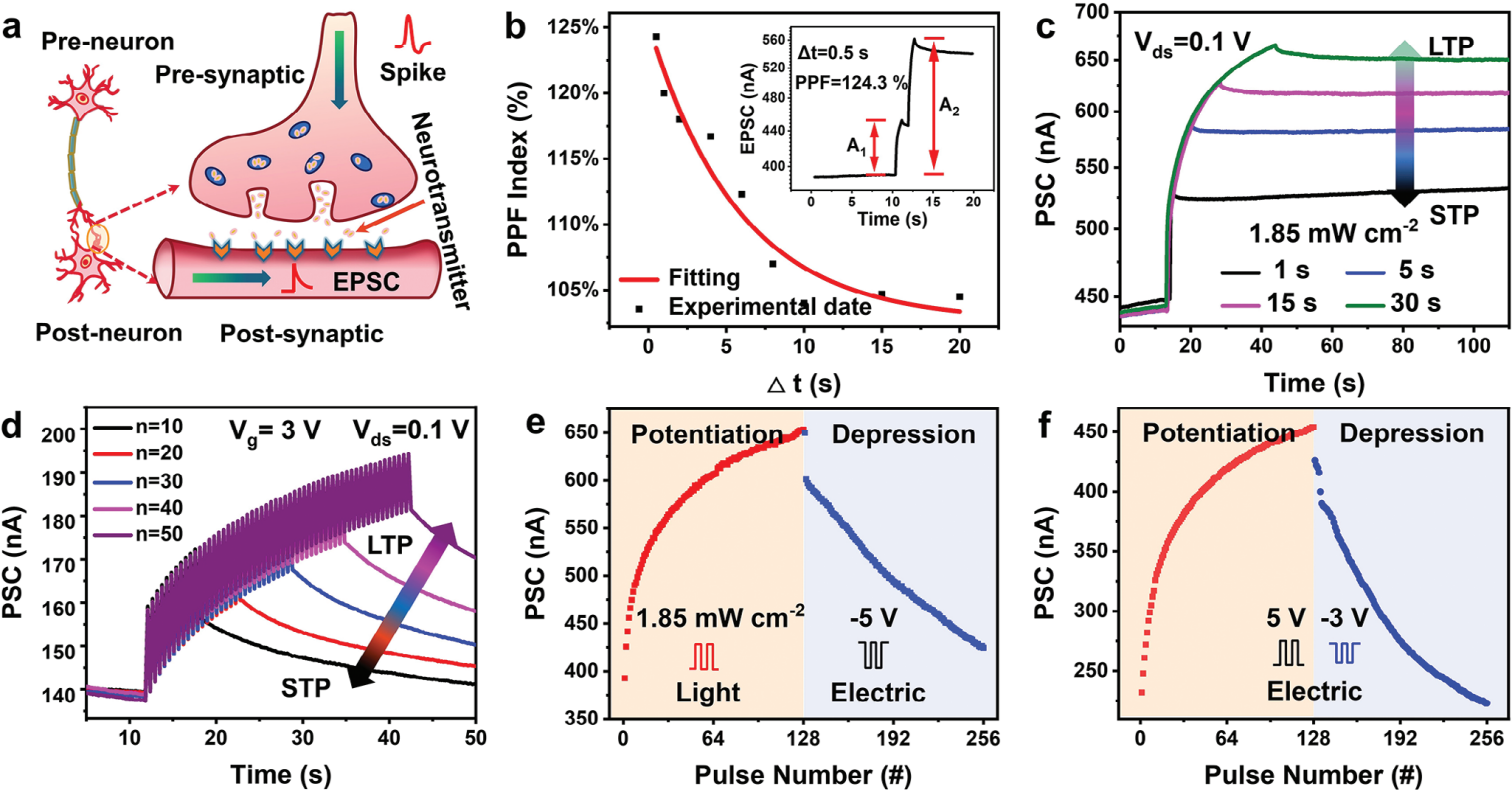
Characterizations of artificial synaptic behaviors in the SnS_2_/h‐BN/CIPS vdWH Fe‐FET. a) Schematic diagram of the biological synapse and transmission of neural signals in a neuron. b) PPF index as a function of the pulse interval time (∆*t*) with a light intensity of 1.85 mW cm^−2^ (the red curve indicates fitting by a double‐exponential decay function). The inset presents PPF triggered by two successive presynaptic spikes with ∆*t* of 0.5 s. The transition from STP to LTP induced by increasing the c) duration of light pulses and d) number of gate voltage pulses. Potentiation and depression process of the synapse mimicked by successive e) optical and negative gate voltage spike pulses and f) positive and negative gate voltage spikes. All PSCs were collected with a read voltage of 0.1 V at the postsynaptic neuron.

Subsequently, we investigated the synaptic plasticity to evaluate the applicability of multilevel memories as artificial synapses. **Figure** **3**a shows a schematic diagram of the biological synapses connecting neurons. Neurotransmitters are released from presynaptic neurons to the postsynaptic receptors under the stimulation of action potentials, triggering excitatory or inhibitory postsynaptic potentials. Therefore, the postsynaptic current (PSC) is considered to be the connection strength between presynaptic and postsynaptic neurons, which is defined as synaptic plasticity. The basic working principle of memory neural networks is based on the synaptic plasticity. The strength or weight of the synapses can be dynamically adjusted from the STP to LTP according to the degree of stimulation.^[^ ^56^ ^]^ In biological neural systems, the PPF behavior is considered a typical feature of STP. This describes the phenomenon in which the PSC generated by the second optical pulse spike is larger than that generated by the first spike and is determined by the pulse interval. Here, the PPF was successfully mimicked by a pair of light stimulus, as shown in Figure 3b. Two continuous optical pulses with different pulse interval times (Δ*t*) were applied to the device, the PSC amplitude induced by the second light pulse was significantly larger than that induced by the first light pulse with a reading voltage of 0.1 V, which is similar to the PPF behavior observed in biological synapses. Furthermore, the PPF index decreases from 124% to 100% as Δ*t* increase, which can be fitted sufficiently by the double exponential function. When Δ*t* was 0.5 s, a maximum PPF value of ≈124% was obtained. Note, a highly resistive state was applied by applying a −5 V voltage before all the synaptic plasticity measurements. By increasing the light pulse time, frequency, and intensity, longer retention times and stronger synaptic weights were observed, which corresponds to the STP‐to‐LTP transition of the memory consolidation process in the human brain (Figure 3c; Figure S10, Supporting Information). The successful simulation of the aforementioned synaptic plasticity was derived from the gradual reversal of light‐induced ferroelectric polarization. In addition, the STP, LTP, and STP‐to‐LTP transitions can also be simulated by applying different electric pulse numbers (n = 10, 20, 30, 40, and 50) and amplitudes (1, 3, and 5 V) (Figure 3d; Figure S11, Supporting Information). Similar to the other two essential synaptic functions, long‐term potentiation and long‐term depression are critical for learning and pattern recognition in artificial vision systems. Figure 3e presents the photonic potentiation and electrical depression behaviors of the SnS_2_/h‐BN/CIPS‐based Fe‐FET. When continuous 128 light pulses (457 nm, 1.85 mW cm^−2^, 0.5 s) were applied to the device, the gradual increase of the channel current showed a potentiation process. Subsequently, the current gradually decreased by applying 128 electrical pulses (amplitude: −5 V, width and interval: 0.1 s), demonstrating a depression behavior. Long‐term potentiation/depression was also emulated by the stimulation of 128 continuous electrical pulses (amplitudes: +5 V; −3 V; width and interval: 0.1 s) (Figure 3f). In summary, the synaptic function simulation can be implemented in two modes including “electrical programming‐electrical erasing” and “optical programming‐electrical erasing”, wherein the programming and erasing of optoelectronic synergistic are critical for flexible learning, adaptation emulation, and pattern recognition in neural networks.

**Figure 4:**
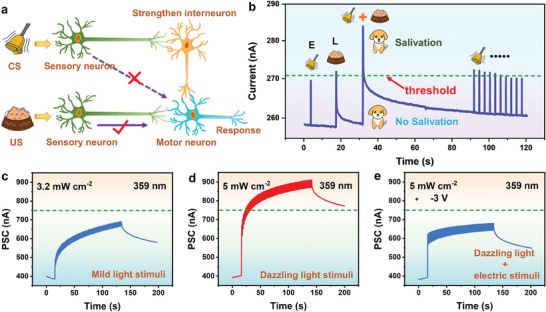
Simulation of associative learning and light adaptation in the SnS_2_/h‐BN/CIPS vdWHs Fe‐FET. a) Schematic diagram of the neural circuitry for associative learning. b) Simulation of associative learning and extinction by the light pulse (0.3 mW cm^−2^, ∆*t* = 0.1 s, 457 nm) and electric pulse (5 V, ∆*t* = 0.1 s). c) Under a mild light stimulation, the photocurrent is below the threshold and does not cause eye damage. d) Under a dazzling light stimulation, the photocurrent exceeds the threshold and causes damage to the eyes. e) Adaptation behavior for the dazzling light stimulation by applying a negative voltage pulse (−3 V) leading to a photocurrent below the threshold. All currents were read at a voltage of 0.1 V.

Pavlovian conditioning, also known as associative learning, is an unconscious memory in which thoughts and experiences complement one another.^[^ ^57^, ^58^, ^59^ ^]^ In Pavlov's dog experiments, bell ringing is a neutral stimulus/conditioned stimulus (NS/CS) that does not initially produce an unconditioned response (UR), and food is considered an unconditioned stimulus (US) that produces an UR, that is, salivation. After training with feeding and bell ringing, an association between NS and US was established, and Pavlov's dogs began to secrete saliva in response to the bell. **Figure** **4**a illustrates a schematic of the receivable neural circuitry for classical conditioning behavior, consisting of sensory neurons (used as input signals), interneurons, motor neurons, and synapses. According to the aforementioned description, the famous Pavlov dog experiment was confirmed for the SnS_2_/h‐BN/CIPS vdWH Fe‐FET (Figure 4b). Light pulses (L) were regarded as food (US) to induce salivary secretion (UR), whereas electrical pulses (E) were used to mimic a bell (CS) to trigger a conditioned response. Here, a PSC weight of 270.5 nA was defined as the threshold for the salivation response. Before training, one electrical pulse (5 V, ∆*t* = 0.1s) was applied to mimic bell ringing, the PSC weight eventually remained below the threshold, indicating that it did not cause the UR. Subsequently, one light pulse (0.5 mW cm^−2^, ∆*t* = 0.1 s, 457 nm) was applied to the device, the PSC weight increased to 271 nA exceeding the threshold, indicating that the UR was finally triggered. Next, light and electrical pulses were simultaneously applied to establish an association between the food and bell; the PSC was above the threshold. Notably, when the light was removed, the PSC triggered by the same electrical stimulation remained higher than the threshold, indicating that cooperative training can promote the conversion from CS to US. Subsequently, electrical stimulation was consecutively applied, and the PSC gradually decreased below the threshold, that is, extinction was achieved, which coincided with the forgetting process in the human nervous system. Furthermore, inspired by the human eye, light adaptation was also simulated by photoelectric collaboration in the vdWH Fe‐FET. In visual adaptation, when the human retina perceives external light signals, the photosensitivity of the photoreceptors (i.e., optic rod cells and cone cells) is adjusted according to the intensity of the signal^[^ ^60^, ^61^, ^62^ ^]^ to allow the eyes to gradually adapt to different levels of light stimulation and avoid damage under bright light. As shown in Figure 4c, the device photocurrent could not reach the threshold of 0.75 µA under a low intensity light stimulation (359 nm, 0.5 s, 3.2 mW cm^−2^), which resembles the situation in which the eyes do not demonstrate discomfort when exposed to mild light stimulation. When the light intensity was increased to 5 mW cm^−2^, the photocurrent increased to 0.9 µA, exceeding the threshold (0.75 µA) (Figure 4d). Subsequently, by introducing a negative voltage pulse (−3 V), the photocurrent was reduced below the threshold under a dazzling light stimulation (5 mW cm^−2^) (Figure 4e). This process is similar to that of artificial retinas, which can adapt to bright light stimulation by adjusting the synapses. In addition, light adaptation was simulated using visible light (Figure S12, Supporting Information). The aforementioned results highlight Pavlovian conditioning, and the broad‐spectrum (UV–vis) adaptation can be simulated successfully by utilizing optoelectronic ferroelectric polarization switching in the synaptic device.

**Figure 5:**
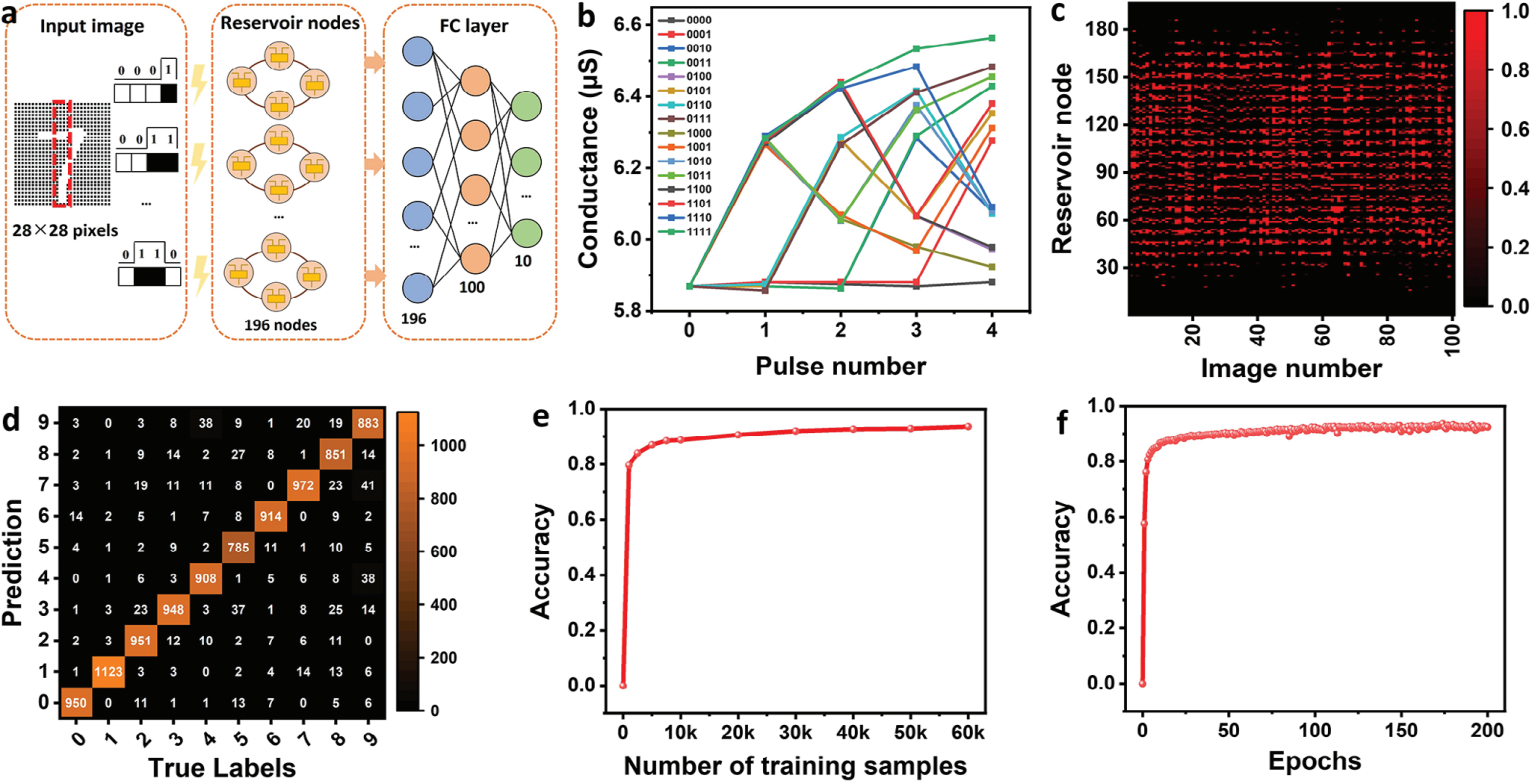
Optoelectronic reservoir computing fully based on the ferroelectric transistors for handwritten digital recognition. a) The architecture and flowchart of reservoir computing for handwritten digital recognition. b) A total of 16 distinct conductance states with valid time series encodings ranging from 0000 to 1111. These extracted features are then fed into the ferroelectric transistors with non‐volatile features to implement fully connected layers for image recognition tasks. c) Conductance states of the randomly selected 100 reservoir images. d) Confusion matrix of recognition results. e) Relationship between the image recognition accuracy and number of training images. f) Variations in the recognition accuracy as the number of training iterations increased.

Considering the nonlinear optical response and multilevel storage characteristics of optoelectronic vdWH Fe‐FET devices, which are beneficial for image recognition using a RC system, we attempted to build an architecture for RC system and performed image recognition. The RC system is a machine‐learning platform for processing temporal information with simple, efficient, and low‐cost training characteristics.^[^ ^7^, ^8^, ^63^ ^]^ It is composed of a reservoir for nonlinear mapping inputs into a high‐dimensional space, followed by a readout network that can be trained to further process the high‐dimensional states.^[^ ^64^ ^]^ **Figure** **5**a presents the processing flow and overall architecture of the optoelectronic reservoir computing system, which consists of a reservoir layer and a fully connected layer fully implemented with SnS_2_/h‐BN/CIPS Fe‐FETs. The reservoir layer adopts Fe‐FETs with optical STP features, which exhibits optical pulse stimuli‐dependent current relaxation dynamics, whereas the fully connected layer is implemented with Fe‐FETs with electrical long‐term potentiation and depression features as synapses to implement the fully connected layer, which is responsible for training and classification. The pixel information of the 28 × 28 MNIST handwritten digit images are first binarized into “0” and “1” through a mask layer. In the RC system, the reservoir layer includes 196 ferroelectric transistors with optical STP features, in which one ferroelectric transistor receives an optical pulse sequence consisting of four successive optical pulses from four pixels in the image. In the optical pulse sequence, “0” represents an optical pulse with an intensity of 0 mW cm^−2^, while “1” represents an optical pulse with an intensity of 1.85 mW cm^−2^ (pulse width 0.5 s). Based on the optical STP characteristics of the device, 16 different conductance states encoded from 0000 to 1111 optical inputs are shown in Figure 5b. After all the 4‐pulse sequences were input into the physical reservoir nodes, the conductance states of the reservoir nodes were read, resulting in 196 reservoir node states for each image. The statuses of 100 randomly selected images are shown in Figure 5c. Subsequently, 196 reservoir states were fed into the fully connected layer with a 196‐100‐10 structure to perform image classification. The synapses in the fully connected layer were implemented according to the long‐term potentiation and depression characteristics of the device. The classification results are shown in Figure 5d. Figure 5e presents the relationship between the image recognition accuracy and the number of training images. The image recognition accuracy increased from 79.65% to 93.62% when the number of training dataset images increased from 1000 to 60 000. Figure 5f demonstrates that the image recognition accuracy increases as the number of training iterations increases, eventually reaching a high accuracy of 93.62%.

## 3 Conclusion

In summary, we reported a novel multi‐functional neuromorphic computing device based on a SnS_2_/BN/CIPS vdWH Fe‐FET. A high‐performance memory and multi‐functional neuromorphic vision system with integrated sensing‐memory‐computing were integrated into a single Fe‐FET for the first time. The device exhibited a high on/off current ratio of over 10^5^, stable cyclic endurance (>350 cycles), long retention time of >10^4^ s, and 128 multilevel current states (7‐bit). Meanwhile, the dynamic synaptic plasticity of PPF, STP, LTP, long‐term potentiation, and long‐term depression were successfully simulated, and retina‐like light adaptation as well as Pavlovian conditioning were demonstrated using the synergistic photoelectric effects. The reservoir layer was implemented by utilizing the optical STP characteristics, whereas the fully connected layer was achieved using the electrical long‐term potentiation and depression characteristics. An image recognition application based on reservoir computing achieved a recognition accuracy of 93.62%. This study provides a promising opportunity for emerging and compact neuromorphic hybrid optoelectronic systems, which is expected to be applied in intelligent robots, driverless technology, and other AI fields.

## 4 Experimental Section

### Device Fabrication

The Cr/Au (10 nm/50 nm) metal electrodes were pre‐deposited on SiO_2_(285 nm)/Si substrate using photolithography and electron‐beam evaporation. The CIPS, h‐BN, and SnS_2_ nanosheets (Nanjing MKNANO Tech. Co., Ltd) were exfoliated on polydimethylsiloxane (PDMS) films, and then were sequentially transferred onto the target electrodes using the dry transfer technique.

### Materials and Device Characterizations

The morphology of SnS_2_/BN/CIPS based Fe‐FET device was characterized by optical microscopy (AOSVI, L100‐HK830), The Raman spectra of nanosheets were obtained by Raman spectrometer (Horiba Labarum HR Evolution, 532 nm). The UV‐vis‐NIR absorption spectrometer (Metatest, MStarter ABS) was used to characterize the band gap of SnS_2_. The STEM specimens were prepared on a FEI Helios 450S dual beam focus ion beam (FIB) workstation, and the cross‐sectional high‐resolution TEM image of SnS_2_/h‐BN/CIPS heterostructure device was obtained by aberration corrected FEI Titan Themis 200. AFM (Bruker Dimension Icon) and PFM mode were used to measure the thicknesses and ferroelectric properties, respectively. The light sources of different wavelengths were provided by commercially available light‐emitting diode (LED) lamps, whose power densities were measured by FieldMate+PM150X photometer. The electronic and optoelectronic measurements were conducted on a vacuum probe station (TTPX, Lakeshore) using a Keithley 4200SCS semiconductor parameter analyzer.

## Conflict of Interest

The authors declare no conflict of interest.

## Supporting information

Supporting Information

## Data Availability

The data that support the findings of this study are available from the corresponding author upon reasonable request.
