# Supplementary material for: Integrated In‐Memory Sensor and Computing of Artificial Vision Based on Full‐vdW Optoelectronic Ferroelectric Field‐Effect Transistor
Source: Adv Sci (Weinh). 2023 Nov 29;11(3):2305679. doi: 10.1002/advs.202305679 (PMC10797471; doi:10.1002/advs.202305679)
Supplement: Supplementary file 1 — Supporting Information [file ADVS-11-2305679-s001.pdf]

ADVANCED SCIENCE
Open Access

Supporting Information

for *Adv. Sci.*, DOI 10.1002/advs.202305679

Integrated In-Memory Sensor and Computing of Artificial Vision Based on Full-vdW Optoelectronic Ferroelectric Field-Effect Transistor

Peng Wang, Jie Li, Wuhong Xue, Wenjuan Ci, Fengxian Jiang, Lei Shi, Feichi Zhou*, Peng Zhou* and Xiaohong Xu**

Supporting Information

Integrated In-Memory Sensor and Computing of Artificial Vision Based on full-vdW Optoelectronic Ferroelectric Field-Effect Transistor

Peng Wang, Jie Li, Wuhong Xue, Wenjuan Ci, Fengxian Jiang, Lei Shi, Feichi Zhou*, Peng Zhou*, and Xiaohong Xu**

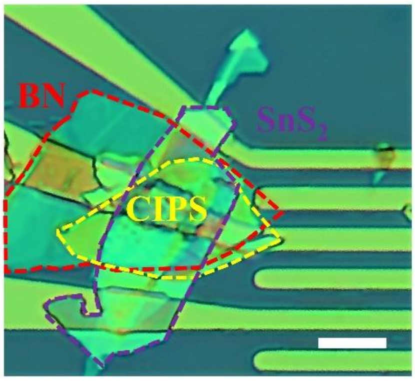

Figure S1. Optical image of SnS_2/h-BN/CIPS based Fe-FET. The lateral dimensions of the CIPS, BN, and SnS_2 nanoflakes are approximately 26, 30, and 55 μm, respectively. Scale bar: 15 μm.

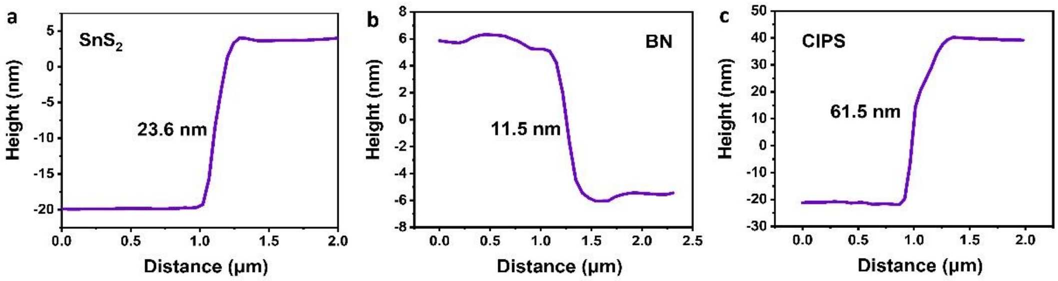

Figure S2. The thicknesses of the exfoliated SnS_2, BN, and CIPS nanosheets are approximately 23.6, 11.5, and 61.5 nm, respectively.

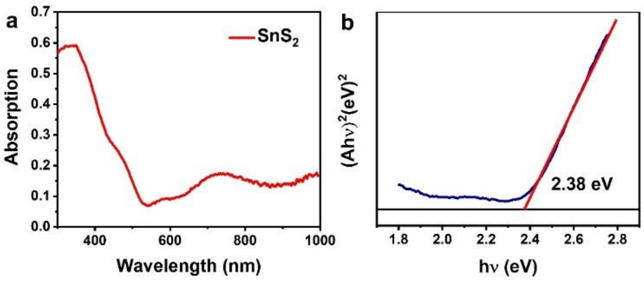

Figure S3. a) The UV-vis-NIR absorption spectrum of SnS_2 nanosheet of ~20 nm transferred onto the mica substrate. b) $(\alpha h\nu)^2$ as a function of photon energy, where α is the absorption coefficient, and the corresponding indirect band gap of SnS_2 nanosheet is 2.38 eV by extrapolating the intercept of the straight line.

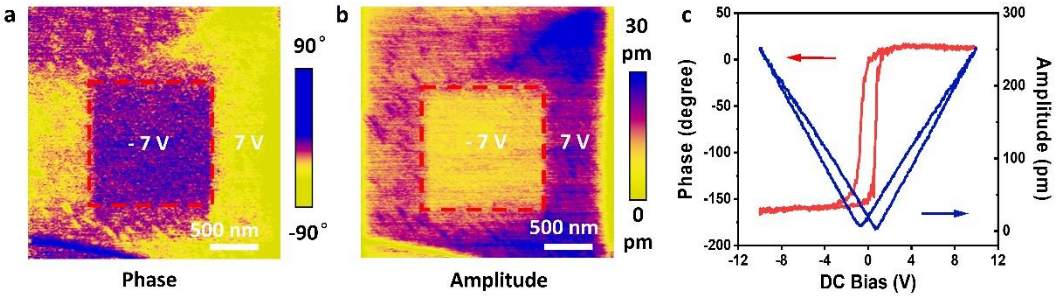

Figure S4. Ferroelectric Properties of CIPS nanosheets. a) Phase and b) amplitude images of CIPS nanosheets at poling of ±7 V. c) The local phase (red) and amplitude (blue) hysteresis loops.

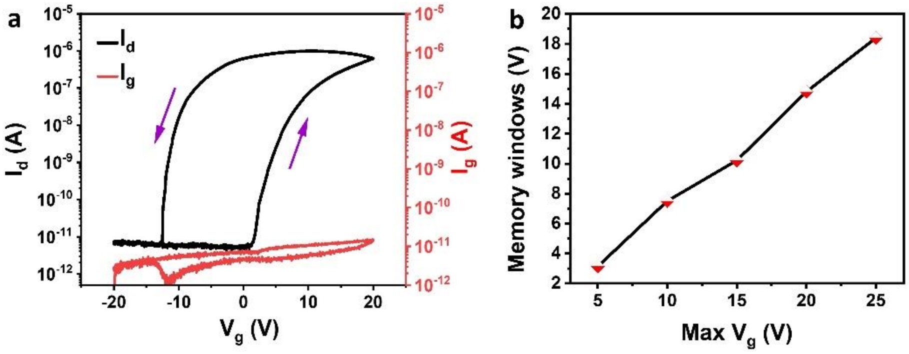

Figure S5. a) The transfer and leakage current curves of SnS_2/h-BN/CIPS based Fe-FET within the scanning voltage range of ± 20 V, V_d = 0.1 V. b) The memory windows of the device versus

different maximum gate voltages with a linear relationship.

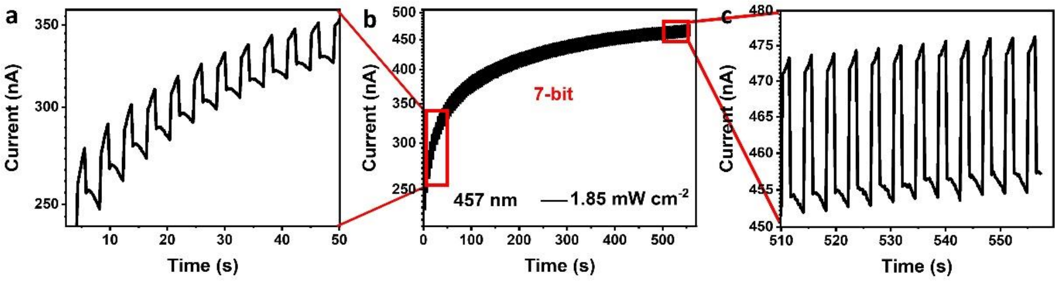

Figure S6. b) The photocurrent under the multiple optical pulses (457 nm, 1.85 mW cm^{-2}, 1.5 s), exhibiting 128 storage states. a,c) The enlarged image of the partial curves in b.

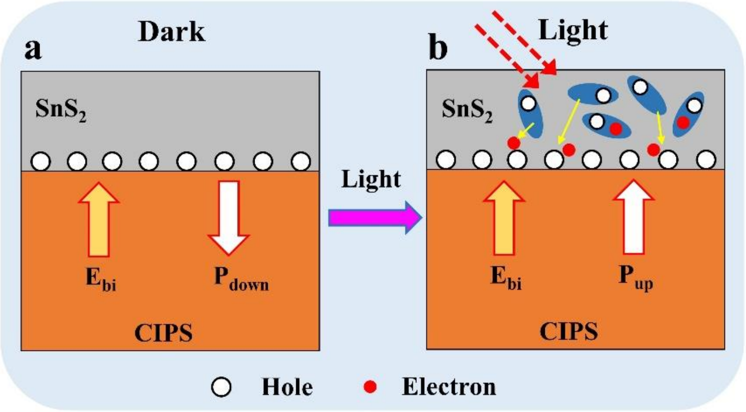

Figure S7. Mechanism of light-induced polarization reversal.

According to the recently proposed theory,[S1-S3] the light-controlled ferroelectric polarization switching can be explained by the interaction between the photogenerated charges in SnS_2 and the ferroelectric polarization charges in CIPS. Due to the fact that the SnS_2/CIPS heterojunction is essentially asymmetric with a preferential upward polarization, which is equivalent to the presence of an upward built-in field (E_{bi}). When the CIPS is in a downward-polarized state, the photogenerated charges under optical illumination compensate for the interfacial charge, leading to upward polarization reversal via the E_{bi}. The ferroelectric polarization switching process strongly depends on the light pulse duration and intensity, and the multilevel storage

following each light pulse sequence can be attributed to the light-induced generation of mixed ferroelectric domain states with different ratios of upward and downward ferroelectric domains.

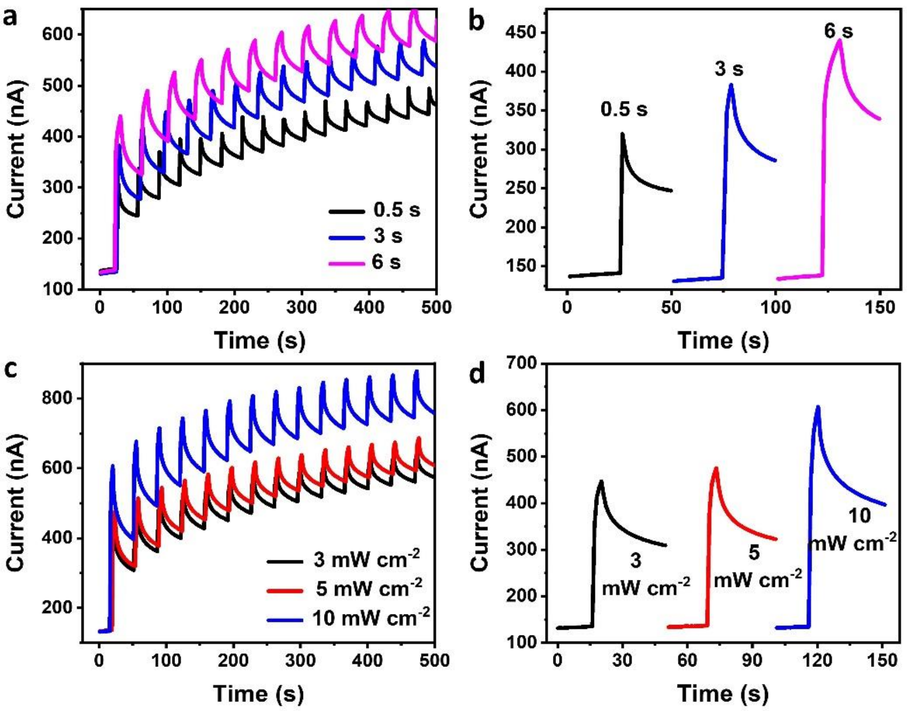

Figure S8. Multilevel storage performance of the device under the light stimulation. Time-dependent output current under light pulses with different a) exposure times and c) light intensity after polarization with -10 V gate voltage. b,d) Photocurrent characteristics of the first pulse in a and c. $V_{read} = 0.1$ V.

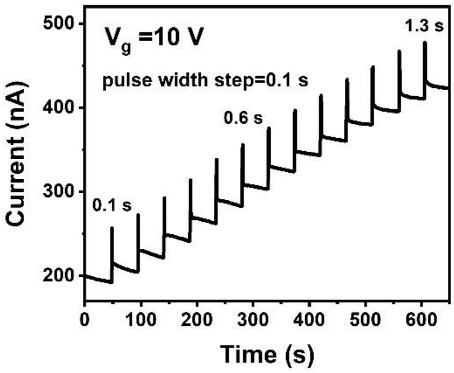

Figure S9. Time-dependent output current under electrical pulses (10 V) with different pulse width from 0.1 to 1.3 s (step: 0.1 s). $V_{read} = 0.1$ V.

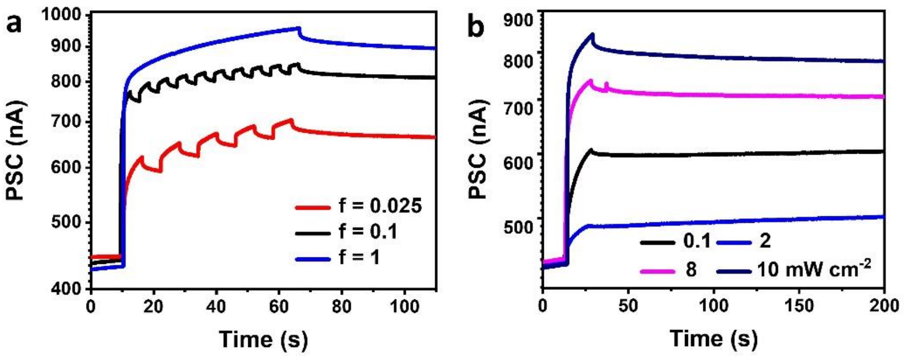

Figure S10. The transition from STP to LTP under different a) frequencies and b) intensities of light pulse. V_{read} = 0.1 V.

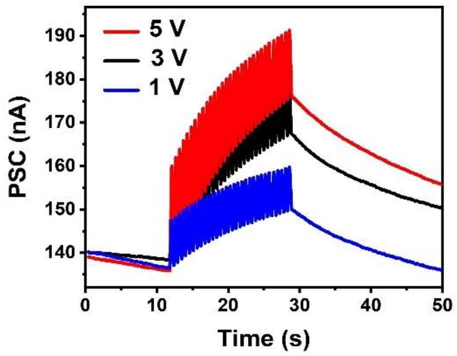

Figure S11. The transition from STP to LTP under pulse voltage of different amplitudes. V_{read} = 0.1 V

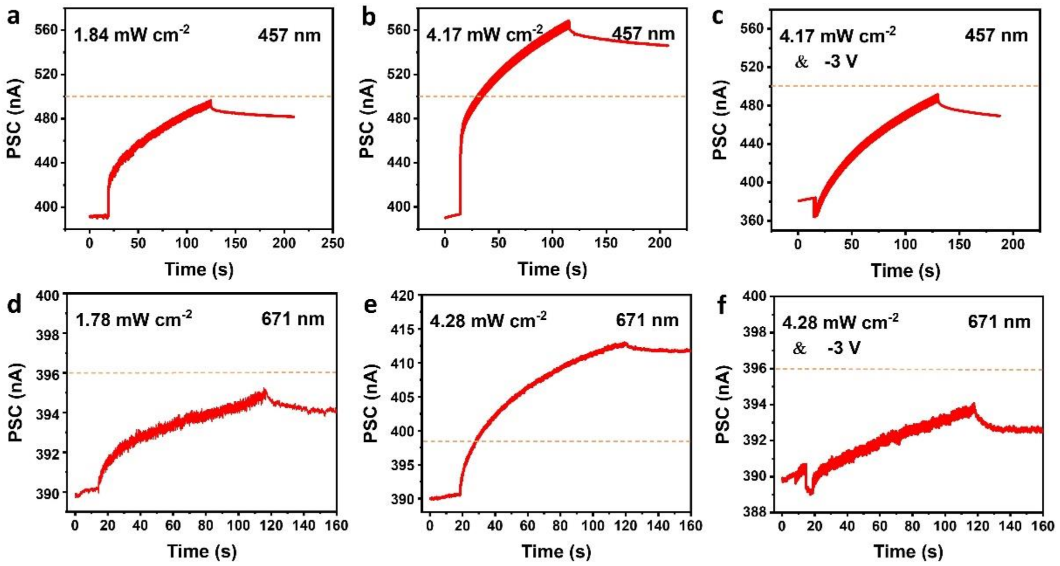

Figure S12. Light adaptation emulation by applying visible lights of 457 and 671 nm, respectively. V_{read} = 0.1 V.

References

[S1] Z. Chen, W. Li, Z. Fan, S. Dong, Y. Chen, M. Qin, M. Zeng, X. Lu, G. Zhou, X. Gao, J. M. Liu, *Nat. Commun.* **2023**, *14*, 3585.

[S2] Z. D. Luo, X. Xia, M. M. Yang, N. R. Wilson, A. Gruverman, M. Alexe, *ACS Nano* **2020**, *14*, 746.

[S3] T. Li, A. Lipatov, H. Lu, H. Lee, J. W. Lee, E. Torun, L. Wirtz, C. B. Eom, J. Iniguez, A. Sinitskii, A. Gruverman, *Nat. Commun.* **2018**, *9*, 3344.
